# Percutaneous left atrial appendage occlusion following failed surgical ligation for incessant left atrial appendage tachycardia: key role of multimodality imaging

**DOI:** 10.1007/s10554-025-03544-1

**Published:** 2025-10-23

**Authors:** Joseph Okafor, Abdulla Mohammed, Balrik Kailey, Alfa Ali, Prapakaran Kanagaratnam, Roberto Casula, Bushra Rana

**Affiliations:** 1 Department of Cardiology, Hammersmith Hospital, Imperial College Healthcare NHS Foundation Trust, London, UK; 2 National Heart & Lung Institute, Imperial College, London, UK; 3 Department of Cardiothoracic Surgery, Hammersmith Hospital, Imperial College Healthcare NHS Foundation Trust, London, UK

**Keywords:** Left atrial appendage occlusion, AtriClip, Multimodality imaging, Echocardiography, Cardiac computed tomography

## Abstract

We describe the case of a 31-year-old male with incessant left atrial appendage tachycardia and resultant severe left ventricular systolic dysfunction. Despite medical therapy, catheter ablation and surgical excision of the left atrial appendage with the AtriClip device, the tachycardia persistent. Following detailed multimodality workup including computational modelling with recreation of virtual implantation scenarios, the tachycardia was eventually terminated with pulsed field ablation and percutaneous left atrial appendage occlusion.

## History of presentation

A 31-year-old-man presented to our institution with chest pain and 6 months of worsening palpitations. He had no prior clinical diagnoses, took no regular medication and was generally fit and active. However, in the past 6 months he had noticed high heart rates on his smartwatch. Family history was notable on the maternal side for a history of heart failure and septal defects in 3 separate relatives.

**Fig. 1:**
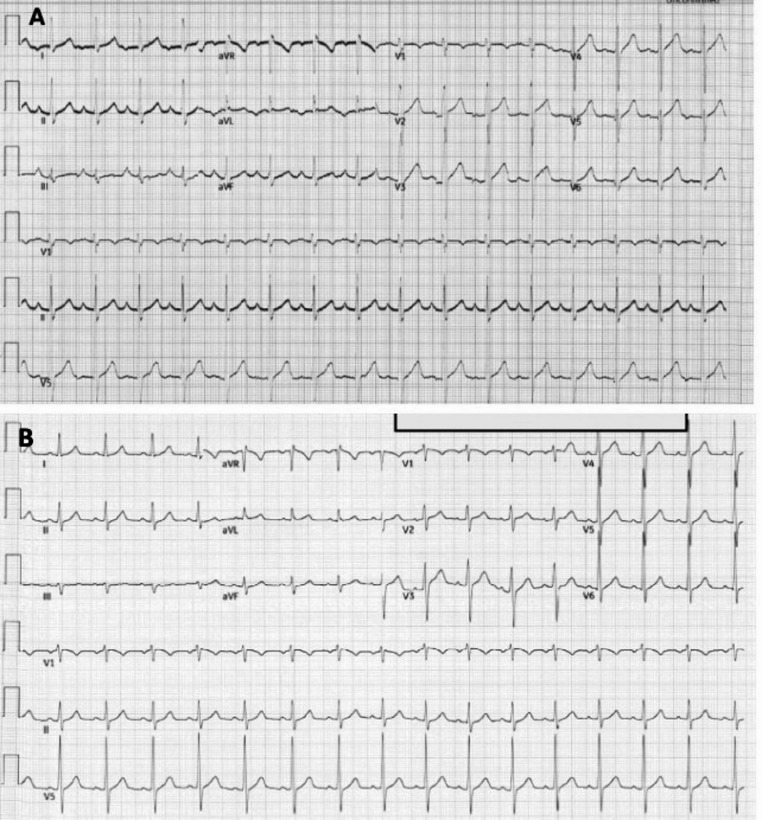
(**A**) ECG of atrial tachycardia. The negative p-waves in leads 1 and aVL can be appreciated, consisted with a LAA origin. (**B**) 6 months follow-up after successful final ablation and LAAO. The ECG p wave morphology is no longer abnormal

Initial 12-lead electrocardiogram (ECG) demonstrated narrow complex tachycardia at 140 beats/min with p wave inversion in lead I and aVL and positive deflection in the inferior leads. P wave morphology suggested atrial tachycardia (AT) with focus from the left atrial appendage (LAA) (Fig. 1A**)**. Initial bedside echocardiography demonstrated severe global left ventricular (LV) systolic dysfunction. Initial treatment with adenosine resulted in a brief slowing of the atrial rate but no termination, while Verapamil bolus temporarily restored sinus rhythm followed by prompt recurrence. At this stage the patient was transferred to our cardiac centre.

On arrival he was persistently tachycardic with heart rate of 142 beats/min, blood pressure of 111/70 mmHg and oxygen saturations of 99% on room air. His lung fields were clear to auscultation, and no heart murmurs were heard. His laboratory workup demonstrated unremarkable full blood count and biochemistry including a normal troponin, C-reactive protein and thyroid function tests. His NT-proBNP was normal at 166 ng/L. Transthoracic echocardiography (TTE) confirmed a dilated LV cavity with severely impaired systolic function, LV ejection fraction (LVEF) of 20–25% and structurally normal valves. The right ventricle (RV) was also impaired with fractional area change of 26%. This was corroborated by cardiac magnetic resonance imaging which revealed biventricular impairment (LVEF 20%, RVEF 46%) without regional wall motion abnormalities. Importantly there was no evidence to suggest a concomitant myocardial disorder since tissue characterisation demonstrated homogonous T1 and T2 mapping signals and no late gadolinium enhancement was seen. Management included guideline directed optimal heart failure therapy.

Despite treatment with verapamil he remained in persistent tachycardia and therefore on day 4, underwent endocardial catheter ablation. The left atrium (LA) electro-anatomical mapping showed the earliest site of activation to originate from the distal region (mid-tip) of the LAA. Careful ablation initially terminated the tachycardia but early recurrence was seen after 15 s. The LA was remapped and a focus identified within the anterior aspect of the LAA base. Further ablation here terminated the tachycardia with no recurrence after 30 min.

However, a few hours after the ablation the tachycardia returned. Ivabridine was introduced and titrated to 7.5 mg twice daily with the aim of controlling the rate to allow LV recovery. Tachycardia persisted albeit at a slowed rate of 100/min and his LVEF remained impaired at LVEF 27%. On day 10, repeat ablation of the distal LAA tip was performed with TEE and CARTO (Biosense Webster, Johnson & Johnson, Irvine CA, US) guidance. Consolidate lesions with eventual bump termination were delivered with various permutations. Following the final lesion, sinus rhythm was acutely restored. Unfortunately, post-procedure atrial tachycardia returned. Amiodarone was then introduced, and the patient was referred for surgical LAA exclusion.

An Atriclip device (AtriCure, Inc, West Chester OH, US) was placed thoracoscopically which resulted in immediate cessation of the AT. Disappointingly, the tachycardia returned in the early post-operative period.

**Fig. 2:**
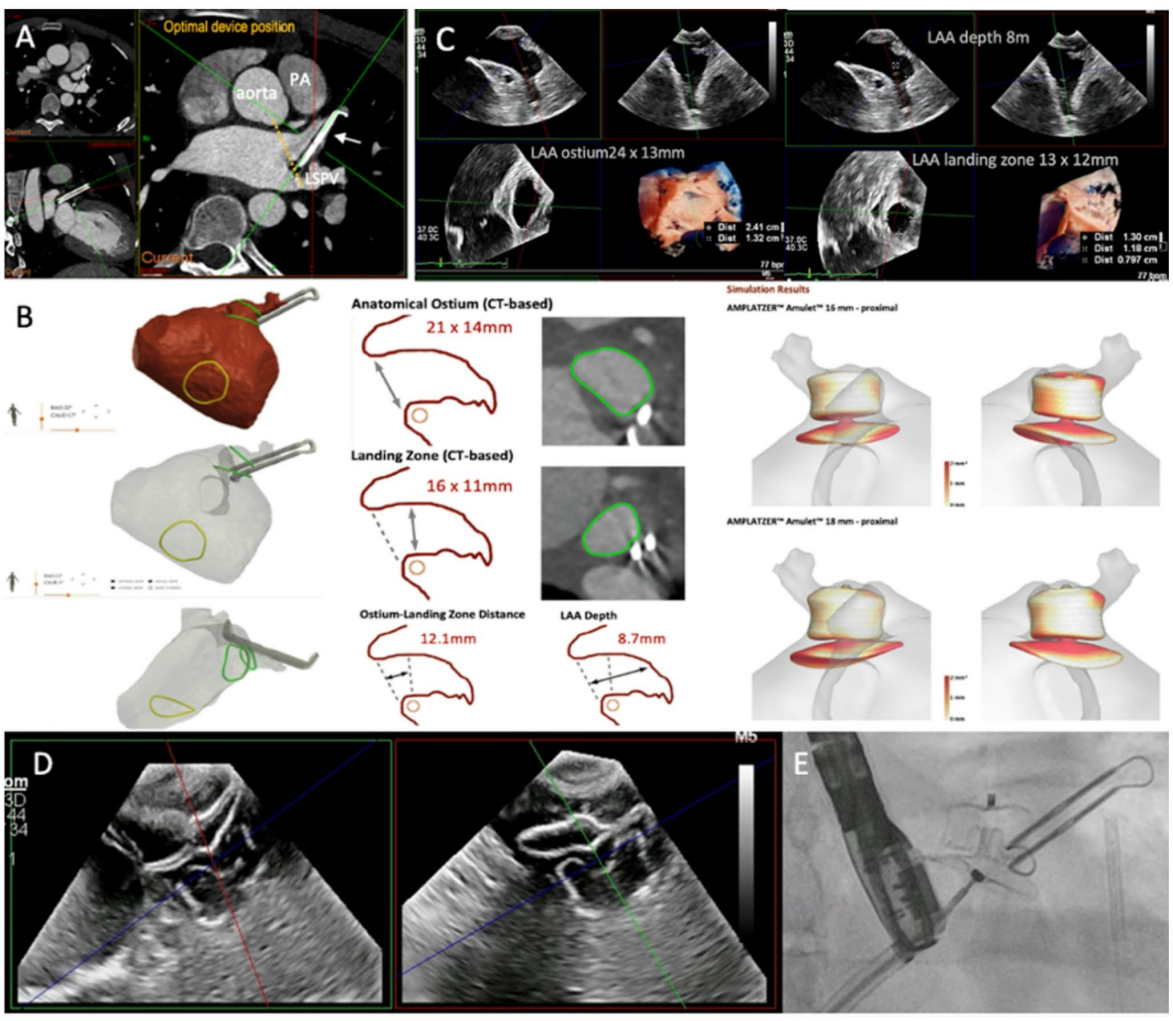
(**A**) delayed phase contrast CT 3D multiplane reconstruction of the LAA following surgical AtriClip placement (white arrow). The AtriClip device can be seen to be displaced and contrast opacifies the whole residual appendage. The yellow dotted line depicts the optimal position for the AtriClip for correct coaxial alignment with the LAA orifice (base of LAA). *PA* pulmonary artery, *LSPV* left superior pulmonary vein. (**B**) FEops CT 3D reconstruction showing the relationship of the AtriClip to the LAA and LA, the green circles show the orifice and landing zone measurements for the Amulet LAAO device. The yellow circle depicts the position of the fossa ovalis. Computational modelling depicts optimal LAAO device size and positioning. (**C**) shows the 3D TEE multiplane reconstruction of the residual LAA with measurements of the LAA orifice, landing zone and depth. These measurements corresponding to the same device choice as the predicted FEops simulation, 18 mm Amulet. (**D**) X-plane image of the 18 mm Amulet LAAO device after deployment at the time of the final procedure. (**E**) Fluoroscopic image of the LAAO device and its relationship to the AtriClip

Following further multidisciplinary team discussions a detailed assessment of the LAA and surrounding anatomy was performed with delayed phase contrast computed tomography (CT). This demonstrated suboptimal positioning of the Atriclip (Fig. 2A**)**. Contrast opacification of the residual LAA lobe was noted all the way to the distal tip. CT analysis to define the dimensions of LAA orifice, the device landing zone and available depth, along with computational modelling for optimal sizing for a virtual LAA occluder (LAAO) device, in this case the Amplatzer™ Amulet™ LAAO (Abbott, Santa Clara CA, US) was performed using FEops Heartguide Platform workflow (Fig. 2B**)**. Therefore, it was felt a further attempt at percutaneous LAA ablation with concomitant LAA occlusion was potentially feasible and should be attempted.

The patient then underwent combined pulsed field ablation (PFA) of the LAA followed by percutaneous LAA occlusion. Using 3D TEE the ostium measured 23 × 13 mm, the landing zone 13 × 12 mm and available LAA lobe depth of 8 mm (Fig. 2C**)**. The 3D transoesophageal echocardiography (TEE) measurements were consistent with the predicted FEops CT derived measurements. An 18 mm Amulet device was selected. The device disc diameter is 24 mm and requires a minimum depth of approximately 10 mm for the compressed lobe length. Therefore the LAAO device lobe might sit a little proud of the orifice. The intention was to fully seal the ostium, and avoid risk of peri-device leak, particularly important in this case as there was high risk of thrombus formation following LAA electrical isolation. Sphere-9™ (Medtronic, Minneapolis MN, US) was used to map the LAA and confirmed distal to proximal activation. After the pulsed field lesions were delivered, sinus rhythm and a heart rate drop was observed. Next, the 18 mm Amulet™ device was delivered via a 12Fr sheath. On the first deployment the device sat well with 22% lobe compression (14 mm compressed diameter), good separation and complete occlusion of the LAA orifice. Using the standard post deployment checks, no peri-device leak was seen. The device stability was confirmed using tug-testing and remained in the satisfactory position following release (Fig. 2D, E**)**.

Six months later, repeat cardiac testing confirmed maintenance of sinus rhythm (also confirmed by no tachycardias recorded on his smartwatch) and normalization of LV systolic function with TTE LVEF 52% (Fig. 1B shows ECG at 6 months). The patient’s symptoms had resolved, and he had returned to regular exercise.

## Discussion

This case details the management of an incessant atrial tachycardia originating from the distal lobe of the LAA complicated by a tachycardia-induced cardiomyopathy. The arrhythmia was resistant to medical therapy (including verapamil, ivabradine and amiodarone) and persisted despite two catheter ablation procedures. The surgical approach failed to achieve full electrical isolation despite immediate AT cessation following LAA lobe exclusion with the AtriClip device. 3D imaging (both 3D TEE and cardiac CT using computational modelling) provided key data in guiding further management. This is the first reported case of combined PFA with concomitant LAA occlusion for AT treatment following failed medical, transvenous and surgical ablation attempts.

AT associated with significant LV impairment is seen more often when the electrical focus originates within the pulmonary vein or LAA. Such AT is often difficult to treat, and medical therapy fails in up to 50% of cases [1]. Alternative strategies including percutaneous ablation should be considered early in the management of such cases [2, 3]. If this strategy fails and where AT focus originates from the distal LAA, surgical LAA excision or exclusion may offer greater success. Surgical appendectomy for treatment of incessant LAA tachycardia has been described in several case reports [4, 5]. In a case series of 42 patients with left or right atrial appendage tachycardia, 30 were successfully managed with radiofrequency ablation while 12 required video-assisted thoracoscopic atrial appendectomy (using a staple with LAA excision) owing to the ablation failure [6]. During appendectomy the tachycardia was terminated immediately after resection of the LAA base.

Successful thoracoscopic LAA exclusion with the AtriClip was first described in 2011 in a 15-yr old male with LAA tachycardia [7]. The AtriClip device is placed over the LAA tip and the body is gradually teased through the clip to position it at the base of the LAA. Device positioning and deployment should ideally be 3D TEE guided to ensure a linear closure line is achieved within the LA. Repositioning is needed if there is residual flow into the LAA, a residual pouch >10 mm or exposed pectinate muscle remain [8]. Post deployment, the correct position is confirmed with TEE at the time of the procedure. At 6-week follow-up imaging using either CT or TEE, device position and persistent complete LAA exclusion are ensured [8]. In our case the AtriClip device was sited, with immediate cessation of the AT and the assumption that complete electrical isolation of the LAA from the LA had been achieved. The recurrence of the AT soon after is likely explained by partial displacement of the AtriClip device. Two major considerations should be assessed prior to such a procedure and underpins the importance of pre-procedure imaging. A delayed phase contrast CT should assess if access to the LAA is possible thoracoscopically, as well as the available space to allow the clip to sit coaxial with the LAA orifice (thereby allowing complete seal of the LAA from the LA cavity). The proximity of the pulmonary artery, left pulmonary veins and aorta may prevent the device resting coaxial at the base of the LAA (LAA orifice level). A long neck with a ninety-degree angle between the plane of the LAA lobe and orifice may leave a residual LAA pouch. An understanding of the risks and recognition of suboptimal deployment by the TEE operator is essential during such procedures. Indeed, to ensure a complete seal has been achieved a linear closure line must be identified at the time of the procedure, prior to device release [8]. In our opinion this can only be fully appreciated using 3D TEE.

In our case, CT assessment of the AtriClip device following recurrence of the AT demonstrated its malposition sitting obliquely across the LAA lobe (Fig. 2A, white arrow) and clearly showed contrast entering the partially patent lobe. The available space to position the device coaxial and at the base of the LAA appeared to be impacted by the position of the aorta, pulmonary artery and left superior pulmonary vein. In order to determine if LAA occlusion was possible in this unique anatomy, the CT data was then uploaded to FEops Heart Guide platform and the residual LAA morphology analysed. Measurements included LAA orifice, landing zone and depth dimensions. Computational modelling created virtual implantation simulations at both proximal and distal implantation depths. This provided an understanding of device behaviour within the residual LAA including device positioning, degree of compression and apposition to surrounding LAA wall (Fig. 2C). The final decision for device size was confirmed at the time of the procedure using 3D TEE measurements which correlated with FEops predicted device size. CT planning allowed accurate LAAO device sizing. 3D appreciation of the complex LAA structure which had been further distorted by the Atriclip was crucial in this case. Furthermore, pre-procedure CT computational modelling was paramount since this ability to model device behaviour and confidently place the optimal device size at the first attempt ensured an efficient and successful procedure.

## Conclusion

In this patient, an incessant LAA tachycardia resulted in severe LV systolic dysfunction. The tachycardia persisted despite medical, catheter ablation and surgical strategies. The tachycardia was eventually terminated with pulsed field ablation and percutaneous LAAO. Peri-procedural planning with CT imaging and in particular computational modelling creating virtual implantation simulations proved pivotal to the eventual success of this case.

## Data Availability

No datasets were generated or analysed during the current study.
